# Porphyromonas gingivalis adopts intricate and unique molecular mechanisms to survive and persist within the host: a critical update

**DOI:** 10.1080/20002297.2020.1801090

**Published:** 2020-08-03

**Authors:** Aditi Chopra, Subraya G. Bhat, Karthik Sivaraman

**Affiliations:** a Manipal College of Dental Sciences, Manipal, Manipal Academy of Higher Education, Manipal, Karnataka, India; b College of Dentistry, Imam Abdul Rahman Faisal University, Dammam, KSA

**Keywords:** Virulence, porphyromonas gingivalis, periodontitis, host, microbe, pathogenesis, inflammation

## Abstract

Porphyromonas. gingivalis (P. gingivalis)

is an obligate, asaccharolytic, gram-negative bacteria commonly associated with increased periodontal and systemic inflammation. *P. gingivalis* is known to survive and persist within the host tissues as it modulates the entire ecosystem by either engineering its environment or modifying the host’s immune response. It interacts with various host receptors and alters signaling pathways of inflammation, complement system, cell cycle, and apoptosis. *P. gingivalis* is even known to induce suicidal cell death of the host and other microbes in its vicinity with the emergence of pathobiont species. Recently, new molecular and immunological mechanisms and virulence factors of *P. gingivalis* that increase its chance of survival and immune evasion within the host have been discovered. Thus, the present paper aims to provide a consolidated update on the new intricate and unique molecular mechanisms and virulence factors of *P. gingivalis* associated with its survival, persistence, and immune evasion within the host.

## Introduction

Periodontitis is a multifactorial immuno-inflammatory disease initiated by the interaction of the host to the pathogenic microorganisms in the oral cavity [1–5]. The host-microbial interaction activates a cascade of signaling pathways that cause release of proinflammatory cytokines and tissue destructive enzymes. The increased inflammatory response in the periodontal tissues alters the composition of the entire microbiome that shifts the gram-positive aerobic cocci to gram-negative anaerobic rods and motile spirochetes [2,6–9]. Some of the common gram-negative anaerobic species that predominate the oral biofilm at the later stages of periodontitis include *Porphyromonas gingivalis (P. gingivalis), Aggregatibacter actinomycetemcomitans* (*A. actinomycetemcomitans), Tanerella forsythia (T. forsythia), Fusobacterium nucleatum (F. nucleatum), Treponema denticola* (*T. denticola), Camplyobacter rectus (C. rectus), Prevotella intermedia (P. intermedia*), etc [3–9]. Amongst all these pathogens, the role of *P. gingivalis* in exaggerating periodontal inflammation and dysbiosis is very unique and intricate [10–17]

*P. gingivalis*, formerly known as *Bacteroides gingivalis*, is a gram-negative non-motile, asaccharolytic, obligate, capnocytophagic, anaerobic rod-shaped bacteria. *P. gingivalis* is considered as a prime etiological agent in the pathogenesis and progression of periodontal inflammation and alveolar bone loss [2,7,12–15]. Approximately, 10%–25% of healthy subjects and 79%–90% of subjects with periodontitis have micro-colonies of *P. gingivalis* in their oral cavity [16,17]. A positive correlation between the depth of the periodontal pocket and the presence of *P. gingivalis* has also been established [17]. Even at low abundance, i.e. <0.01% of the total bacterial count, *P. gingivalis* has the inherent ability to transform the entire microbial community and amplify the disease process [10]. It has been referred to as a ‘keystone pathogen’ and a ‘master of polymicrobial synergy, dysbiosis and immune subversion’, as it exploits several sabotage tactics to evade, weaken, or deceive the host’s immune system [10–15]. *P. gingivalis* is also known to gain entry into the host cell and systemic circulation to reach distant organ systems [15–19]. Numerous studies have explained and discovered the novel virulence factors and pathogenic mechanisms that help *P. gingivalis* to survive and persist in the host [12–15]. However, the knowledge about how *P. gingivalis* modulates the immune response with activation of newly discovered virulence factors, host proteins, and signaling pathways is limited. No paper to our knowledge has provided a consolidated update on the recent immunological receptors, signaling pathways, and molecular and cellular mechanisms associated with *P. gingivalis* invasion and persistence within the host tissue. Furthermore, the knowledge about the cellular and molecular mechanisms linked with the interaction of *P. gingivalis* with other members of the oral microbiome is not fully explored. It is important to understand how *P. gingivalis* causes of suicidal death of other members of the oral microbiome along with the emergence of pathobionts and virulent commensals in the host.

Therefore the present review aims to provide a critical and consolidated update on the new pathogenic mechanisms and survival strategies of *P. gingivalis* within host associated with periodontal inflammation and dysbiosis. A thorough understanding of these key virulence factors and pathogenic mechanisms is crucial not only to understand the pathogenesis of periodontal diseases and systemic inflammation but also to develop novel therapeutic modalities that to prevent the onset and progression of periodontal disease.

## Mechanism of P. gingivalis invasion and persistence within the host

**Figure 1.**
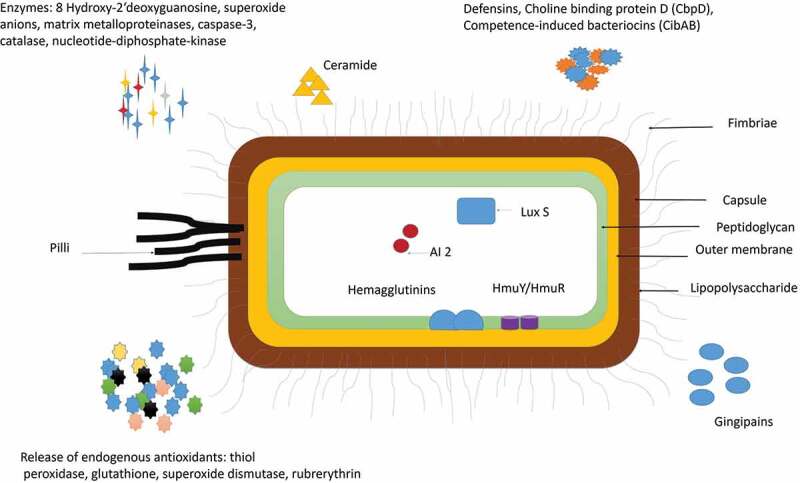
**Virulence factors of P. gingivalis**: P gingivalis has various virulence factors that help it to invade the host cell and evade the defense mechanisms of the host. Some of the most important adhesions of P. gingivalis include Lipopolysaccharide (LPS), Capsule, outer Membrane protein, Peptidoglycan, Major, and minor Fimbriae and Pilli. P. gingivalis also release enzymes and cytotoxic molecules such as defensins, autoinducer proteins (AI-2); 8 hydroxy 2ʹ deoxyguanosine, superoxide anions, matrix metalloproteinases, caspase-3, catalase, nucleotide-diphosphate-kinase. These enzymes help to invade the host cell, increase oxidative stress, and enhance biofilm formation. P. gingivalis is also known to release endogenous antioxidants such as thiol, peroxidase, glutathione, superoxide dismutase, rubrerythrin to protect itself from the surrounding free radicals.

*P. gingivalis* is a virulent subgingival periodontal pathogen that can orchestrate the inflammatory response within the host by altering various inter-bacterial and host-bacterial interactions [10–20]. *P. gingivalis* survives within the host by adhering to a wide range of oral substrates and molecules like extracellular matrix proteins, oral epithelial cells, and other commensal bacteria such as *Streptococci gordonii (S. gordinii), Filifactor alocis (F. alocis), F. nucleatum*, and *Actinomyces viscosus* [21–26]. *P. gingivalis* impairs various components of the innate immune response: alters the functions of the complement system, Toll-like receptors (TLRs), neutrophils, macrophages, dendritic cells, and, T cells (Th1/Th2/Th17) [14–17]. It can even modulate its virulence factors and nutritional demand to survive and persist at the times of crisis and nutritional deficiency [12–23]. Gingipains (Cysteine proteases), capsule, lipopolysaccharide (LPS), fimbriae, nucleoside diphosphate kinase (NDK), ceramide, and outer membrane proteins (OMs) are some of the important virulence factors that help *P. gingivalis* to invade and survive with the periodontal tissues and gain entry into the systemic circulation (Figure 1) [21–25]. The ‘inflammophilic’ nature of *P. gingivalis* even helps it to utilize the tissue destructive byproducts, enzymes, and proinflammatory cytokines produced during inflammation as its nutrients [26–28]. *P. gingivalis* can even indirectly modulate the maturation and growth of other microorganisms within the biofilm growth by indirectly sending the short-range paracrine signaling molecules into its environment and enhancing quorum sensing [3,23,28–30]. The interaction with other microorganisms not only facilitates its growth and survival but also favors the growth and development of other bystanders, commensal, and pathobiont species [29–32]. Some of the other unique and intricate molecular mechanisms associated with its interaction of other members of the biofilm and host that increases its growth, survival, and persistence can be summarized as follows (Table 1):

**Table 1.** Novel Pathogenic mechanisms adopted by *P. gingivalis* to exaggerate periodontal inflammation.

S/No	Pathogenic mechanisms		Reference
1.	Synergistic interaction with other organisms and the development of pathobiont species	Growth of bystander or commensal microbiota The emergence of pathobiont species such as *F. alocis, Peptostreptococcus stomatitis, Prevotella, Megasphaera selenomonas*, and *desulfobulbus* Activation of intracellular nucleotide-binding oligomerization domain 1 (Nod1) A shift of nutrient requirement from protein-dependent to carbohydrate dependent Non-identity-mediated CRISPR-bacteriophage interaction Co-operative haem acquisition by the HmuY haemophore of Porphyromonas gingivalis	Kolenbrander et al., 2006 Hajishengallis et al., 2011 Jiao et al., 2014 Aruni et al., 2014 Hasegawa et al., 2006 Cady et al., 2011dr Byrne et al., 2013
2.	Survival in the host cell and neutrophil dysfunction	Regulation of expression of E-selectin on the endothelial cell surface Binding of FimA to β1 integrin receptor Induces the expression of transposases Release of serine phosphatase protein (SerB) and dephosphorylates the actin-depolymerizing molecule ‘cofilin’ Activates Heat Shock Protein (HSP) and GAPDH (glyceraldehyde 3 phosphate dehydrogenase) receptors Inhibit IL 8, cytokine-induced neutrophil chemoattractant (CINC) 2αβ Induce microRNAs (miR-105 and miR-203).	Reife et al., 2006 Darveau et al., 1995 Duran-Pinedo et al., 2014 Bainbridge et al., 2010 Huang et al., 2001 Takeuchi et al., 2013 Benakanakere et al., 2009
3.	Anti-apoptotic mechanism	Inhibition of Janus A Kinase (JAK), Phosphoinositide 3 Kinase (PI3 K), Signal Transducer of Activation (STAT), alpha-serine/threonine-protein kinase (Akt) and purinoceptor (P2X7 receptors). Activate p38, mitogen-activated protein kinase (MAPK), and extracellular-signal-regulated kinase (Erk1/2) pathways. Decrease expression of cyclin D at the G1 phase. Increase Apoptotic protease activating factor (Apaf 1), B-cell lymphoma Associated X (Bax1) and Caspase 3 production Reduce B-cell lymphoma (BCL 2) expression Regulate NLR family pyrin domain containing 3 (NLRP3) inflammasome expression	Zaric et al., 2010 Choi et al., 2013 Yilmaz et al., 2008 Song et al., 2016 Bugueno et al., 2018 Huck et al., 2015
4.	Increased proinflammatory cytokine production	Binding of LPS to Toll-like receptors (TLRs), Receptor activator of nuclear factor kappa-B ligand (RANKL) and complement receptor (CR) A shift of LPS moiety from Penta acylated to tetra acylated isoform Activation of TLR 2/4 and C5aR on the neutrophil surface LPS induced increase in Thrombospondin 1 (TSP) production and Plasminogen activator inhibitor type 1 expression Down-regulate T-helper 1 (Th 1) cells Activation of Protease-activated receptor (PAR) 2 and soluble triggering receptor (sTREM) Increase production of IL17	Lu et al., 2009 Reife et al., 2006 Gokyu et al., 2014 Na et al., 2014 Bostanci et al., 2013 Nylund et al., 2017 Moutsopoulos et al., 2012
5.	Subversion of the complement system	Inhibits all components of the complement system and degradation of its by-products (C3a and C5a) Prevents formation of membrane attack complex (MAC) by inhibiting Mannose-binding lectin (MBL), Ficolins (FCN), C3, C3b, and C4. Utilizes HRgpA gingipain to entrap the circulating C4b binding protein.	Sundqvist et al., 1985 Potempa et al., 2008 Hajishengallis et al., 2007
6.	Disruption of T cell function	Induce Th1 immune response and increase secretion of IL 1, IL6, IL10 production A decrease in levels of IL12 and increased production of Interferon-alpha Favors Th-17 activation by interaction with dendritic cells of the host	Cardoso et al., 2009 Moutsopoulos et al., 2012 Yoshimatsu et al., 2009
7.	Suppression of macrophage activity	Increase cAMP formation Reduce the expression of inducible nitric oxide synthase (iNOS) Activation of caspase 11 dependent non-canonical inflammasome in the macrophage Altered macrophage function and antigen presentation Attenuate CR3 activation in macrophages, reduce inhibition of lncRNA GAS5, with less miR-21 and more IL12 production. by inhibiting sialidase	Nathan., 2012 Moon et al., 2014 Yung et al., 2018
8.	Modulation of gene expression in the host and other bacteria	Modify levels of hemin, polyphosphate, rhein, polyphosphate, Temperature-dependent modulation of Porphyromonas gingivalis lipid A structure Upregulation of putative transposases in *S. mitis* and *Lactobacillus casei* microcolonies Upregulation of CRISPR RNA and toxin-antitoxin system proteins	Anaya-Bergman et al., 2006; Phillips et al., 2006; Al-Qutub et al., 2006 Curtis et al.,2011a Aruni et al.,2014 Jorth et al., 2012;
9.	Fratricide and altruistic ‘suicide’ of other bacteria	Release of Choline Binding protein D (CbpD), competence-induced bacteriocins (CibAB), Activation of Autolysin-Encoding Gene Activation of bacterial apoptosis endonuclease (BapE)	Duran-Pinedo et al., 2014 Eldholm et al., 2009
10.	Increased production of antioxidant species	Activation of NOX 4 receptors Increase extracellular ATP production by activation of P2X4, P2X7, JAK2, and pannexin-1 receptor Increase production of NADPH oxidase Intracellular release of endogenous antioxidants like thiol peroxidase, glutathione, superoxide dismutase, rubrerythrin	Gölz et al., 2014 Bedard et al., 2007 Wang et al., 2014 Goettsch et al., 2013 Sztukowska et al., 2002 Kikuchi et al., 2005

***2.a. Synergistic interaction of P. gingivalis with other microorganisms and development of pathobiont species***

**Figure 2.**
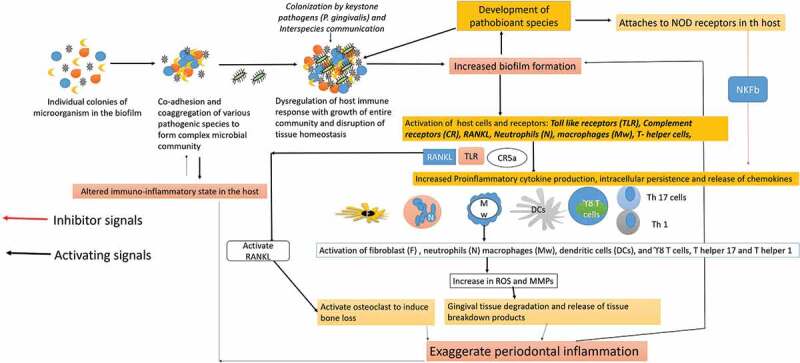
**P. gingivalis modulates the host immune response by facilitating the growth of pathobionts species and altering the function of various immune cells of the host. The weakened immune response enhances biofilm formation and oxidative stress that in turn increases the periodontal inflammation and favors the growth of P. gingivalis** *[Abbreviation: CR- complement receptors; TLR- Toll-like receptors; ROS-Reactive oxygen species; MMPS- matrix metalloproteinase; IL- interleukin; RANKL- Receptor activator of nuclear factor-kappa beta; TNF- Tumor Necrotic Factor-alpha; Th – T helper cells]. [Abbreviation: CR- complement receptors; TLR- Toll-like receptors; ROS-Reactive oxygen species; MMPS- matrix metalloproteinase; IL- Interleukin; RANKL- Receptor activator of nuclear factor-kappa beta; TNF- Tumor Necrotic Factor-alpha; Th – T helper cells; NOD-nucleotide-binding oligomerization domain; NF-Kb – Nuclear Factor kappa Beta].*

**Figure 3.**
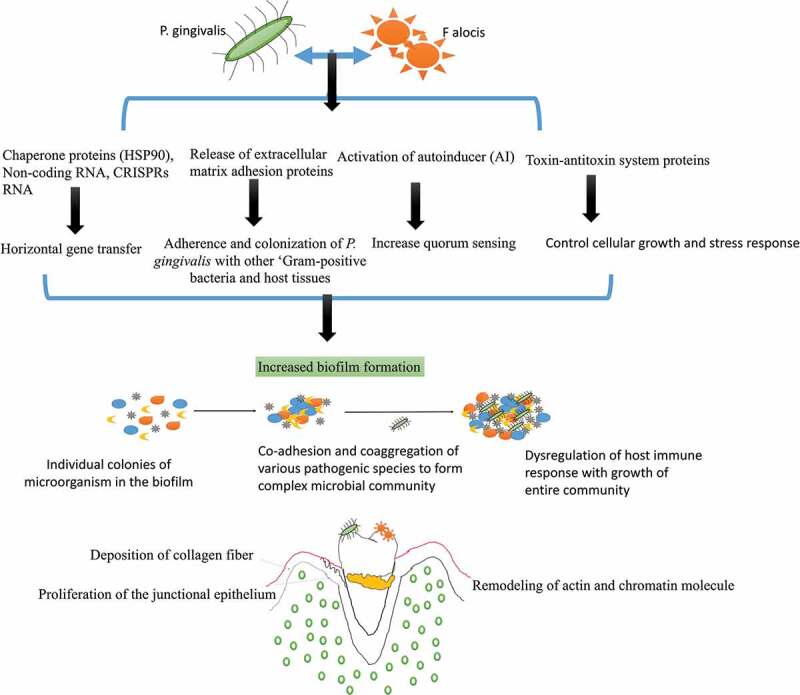
**Interaction of P. gingivalis with F. alocis and its effects on biofilm formation and periodontal inflammation**: P. gingivalis interaction with F. alocis can modulate the innate immune response of the host as both these species auto-aggregate and express unique genes expression. *P. gingivalis-F. alocis* remodeling the actin and chromatin molecule, activation of autoinducer (AI) associated quorum sensing, the proliferation of the junctional epithelium, and deposition of collagen fibers in the gingival epithelial cells. The co-infection also upregulates the production of extracellular matrix adhesion proteins, CRISPRs RNA, and toxin-antitoxin system proteins that increase the adherence and colonization of *P. gingivalis* with other ‘Gram-positive bacteria and host tissues’, and are directly linked with increased biofilm formation. The CRISPR-RNA also helps with triggering the stress response, chaperone formation, and horizontal gene transfer among oral bacteria that favor microbial community development.

**Figure 4.**
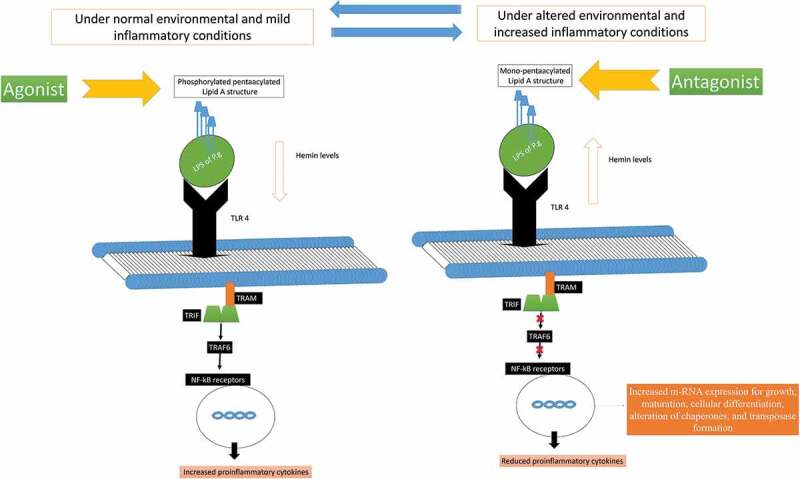
Modulation of immunoinflammatory response by two forms of P. gingivalis lipid A depending on the microenvironment (hemin level) and their interference in TLR4 receptor signaling downstream activation. It can regulate the inflammatory response according to changes in the environmental conditions by changing its LPS moiety *[Abbreviation: LPS: lipopolysaccharide; p65: nuclear factor NF-κB protein p65 subunit; p50: nuclear factor NF-κB protein p50 subunit; TLR4: Toll-like receptor-4; TRAF 6: tumor necrosis factor receptor-associated factor 6; TRIF: TIR-domain-containing adapter-inducing interferon-β; TRAM: TRIF-related adaptor molecule].*

**Figure 5.**
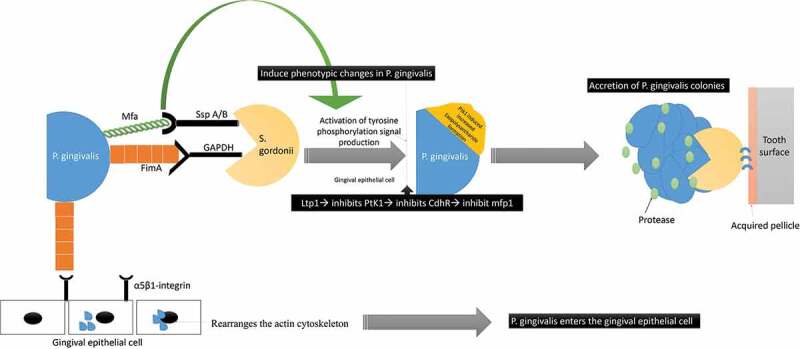
**Schematic representation of the interaction between *P. gingivalis* and S. *gordonii****: P. gingivalis* interacts with *S. gordonii* by utilizing its major (FimA) and minor fimbriae (Mfa1). FimA and Mfa1 of *P. gingivalis* binds to glyceraldehyde‐3‐phosphate dehydrogenase (GAPDH) and streptococcal SspA/B adhesins (SspA/B termed BAR antigen I/II) of *S. gordonii*, respectively The Mfa1 interactions with SspB lead to phenotypic changes with subsequent activation of tyrosine phosphorylation signal production (PTK). The increased PTK signaling causes exopolysaccharide formation that causes accretion of P. gingivalis colonies. The FimA also interacts to the α5β1-integrin receptors on gingival epithelial to facilitate bacterial entry.

*P. gingivalis* interacts with other microorganisms to elicit its full range of pathogenicity and virulence factors, as in germ-free conditions, it cannot survive or produce any disease (Figure 2) [10]. The interspecies synergy of *P. gingivalis* has been observed with *Streptococci mitis (S. mitis), S. gordonii, A. actinomycetomcomitans, F. nucleatum, T. denticola, P. intermedia, F. alocis* [8–10,24,30]. *P. gingivalis* modulates the phenotypic profile of microorganisms by interacting with their surface adhesins and regulating their virulence factors, nutritional demands, and growth [25–28]. The most common synergistic interaction of *P. gingivalis* is observed with *F. nucleatum, S gordonii and F. alocis* (Figure 2–5) [30–37]

Recently, *P. gingivalis’* is discovered to favor the development of many pathobiont species in the oral biofilm. A pathobiont is a harmless symbiont that becomes pathogenic under altered environmental conditions or immunosuppression [15,30–38]. The pathobionts outcompete the other anaerobic species to become the major etiological agent for the onset of periodontitis [23,32,33]. A recent metatranscriptomic and metagenomic analysis has confirmed a positive correlation between *P. gingivalis* and development of several underappreciated pathobionts species like *Peptostreptococcus stomatis, F. alocis, Megasphaera, Selenomonas, and Desulfobulbus* [13,15,30–32]. The pathobiont species help *P. gingivalis* to acquire essential nutrients, enhance virulence factors, and activate the release of cytokines and enzymes in the oral environment [30–34]. The pathobionts even accumulate at the sites of damaged periodontal tissues and selectively bind to the intracellular nucleotide-binding oligomerization domain 1 [Nod1] like receptors that indirectly activates the Nuclear Factor-kappa Beta (NF-Kb) mediated proinflammatory cytokines production (Figure 2) [34–39].

The most common pathobiont that favors *P. gingivalis’* growth and survival is *F. alocis*. Studies have shown that *P. gingivalis* co-cultured with *F. alocis* can modulate the innate immune response of the host as both these species auto-aggregate and express unique gene expression (Figure 3) [31–33]. *P. gingivalis-F. alocis* is linked with the remodeling of actin and chromatin molecule, activation of autoinducer (AI) associated quorum sensing, the proliferation of the junctional epithelium, and deposition of collagen fibers in the gingival epithelial cells [30–33]. The co-infection also upregulate the production of extracellular matrix adhesion proteins like actin, vinculin, vimentin, plectin, transgelin, profilin, endoplasmin proteins (filamin B and filamin C), chaperone proteins (HSP90), non-coding RNA, CRISPRs RNA, ‘microbial surface component-recognizing adhesion matrix molecules’ (MSCRAMMs), and toxin-antitoxin system proteins [31]. These novel proteins are required for the adherence and colonization of *P. gingivalis* with other ‘Gram-positive bacteria and host tissues’, and are directly linked with increased biofilm formation [31–40]. The increased production of CRISPR-RNA is also associated with triggering the stress response, chaperone formation, and horizontal gene transfer among oral bacteria [41–43]. The CRISPRs RNA even helps *P. gingivalis* to induce genomic rearrangements and intercellular recombination that helps to acquire the useful DNA sequences for its survival, limit transposition of insertion sequences and acquire resistance to foreign RNA and DNA [44–48]. Watanabe et al. (2017) also observed a highly ‘expressed transcripts of CRISPR regulatory small non-coding regulatory RNA (sRNA) in the intergenic sequences (IGS) upstream of CRISPR-associated (*cas*) gene arrays in *P. gingivalis* that help to limit the genetic exchange within the biofilm and prevent mutation [42,45–47]. Another highly upregulated hypothetical proteins, known as ‘HMPREF0389_00967, containing a CHASE3 extracellular sensory domain’, has been observed during *F. alocis – P. gingivalis* co-infection [31,48]. The HMPREF0389_00967 is found to regulate the production of histidine kinase, adenylate cyclases, and other chemotaxis proteins that helps *P. gingivalis* to evade the process of phagocytosis [31,45–48]. *F. alocis – P. gingivalis* co-infection is also known to regulate other proteins such as ‘histone cluster proteins and PPIA-peptidyl-prolyl isomerase, transferrin receptor protein 1 (involved in iron transport), transmembrane emp24 domain-containing protein 10, dynein (involved in vesicular protein trafficking), surfeit 4, and solute carrier family proteins’ in the host [48]. The alteration in the production of these proteins indirectly weakens the innate immune response of the host and favors microbial growth within the tissues. *P. gingivalis* can even acquire and utilize some of these proteins, nucleosides, and nucleobases for its nutrition, growth, maturation, and survival [49].

*P. gingivalis* is known to modulate its nutritional demand and virulence factors and according to the changes in its environment, immune response, co-infection, and temperature and nutrient supply [12,47,50–60]. Studies have confirmed that the addition of *P. gingivalis* to a healthy multispecies oral biofilm model rearranges the DNA profiles of other commensal species, alters the functions of the complement system, subvert phagocytosis, protease production, alter its adhesions, and cause cytotoxicity [12,20,50–54]. *P. gingivalis* also changes the genetic expression of other commensal and symbiotic bacteria according to changes in the level of hemin, polyphosphate, rhein, temperature, oxidative stress, etc. in the periodontal tissues [47,54–59]. Studies have shown that high levels of hemin and rise in environmental temperature cause *P. gingivalis’* to alter its Pentaacylated LPS 1690 isoform to the Tetraacylated LPS 1435/1449 isoform [47,51–59]. This alteration in the LPS structure inhibits the process of phagocytosis by inhibiting the expression of LPS binding proteins (LBP) and the production of key chemoattractants like IL8 and IL6 [53]. High levels of hemin and rise in temperature also upregulate the expression of mRNA associated with the growth, maturation, cellular differentiation, alteration of chaperones, ABC transport systems, and transposases formation (Figure 4) [33,47,55–61]. Duran-Pinedo et al., (2014) observed that *P. gingivalis* invasion upregulate the expression of putative transposases in *S. mitis* and *Lactobacillus casei* microcolonies that enhance biofilm formation [61–68].

*P. gingivalis’* interaction with *S. gordonii* is also confirmed to have a profound effect on the phenotypic profile of the entire microbial community with activation of several genetic functions before the ‘mutualistic communities’ development [21,62–66]. *S. gordonii* facilitates the colonization of *P. gingivalis* even in the absence of any bridging species like *F. nucleatum* [10,61–72]. *P. gingivalis* adherence to *S. gordonii* is by a ‘multimodal’ and ‘coordinated adhesion system’ comprising of the major (FimA) and minor (Mfa1) fimbriae [65–68]. FimA and Mfa1 of *P. gingivalis* bind to glyceraldehyde‐3‐phosphate dehydrogenase (GAPDH) and streptococcal SspA/B adhesins (SspA/B termed BAR antigen I/II) of *S. gordonii*, respectively (Figure 5) [67–74] Moreover, it has been confirmed that community development with *P. gingivalis* does not occur with *Streptococci* species that lacks the BAR motif, such as *S. mutans* and *S. intermedius* [63,64,75]. Therefore, even though, all of the oral streptococci express antigen I/II, *P. gingivalis* selectively binds to *S. gordonii* and the related oralis group of *Streptococci* [76]. Additionally, studies have shown that before *P. gingivalis* adheres to *S. gordonii*, it accretes to form ‘tower-like microcolonies’ that are separated by fluid-filled channels [76,77]. *P. gingivalis – S gordonii* co-infection even favors the ‘heterotypic community formation with *F. nucleatum. P. gingivalis-F. nucleatum* coaggregation is mediated by a galactoside moiety on *P. gingivalis’* surface and a lectin adhesion on *F. nucleatum* [10,16,21,26–29,33]. Studies have observed that *F. nucleatum* increases the invasion of *P. gingivalis* into the gingival epithelial cells by 2–20‐folds, along with an increase in the osteoclastic activity, immune modulation with enhanced proinflammatory cytokine production and apoptosis [34–36]. The adaptation of a community lifestyle provides physiologic support to its constituent species, lower levels of environmental stress, and promotes the chance of survival [75]. *S. gordonii* is considered as a homeostatic commensal that is capable of mitigating the activity of *P. gingivalis* and modulating the host signaling [78–82].

*P. gingivalis-F. nucleatum- S. gordonii* consortia is known to cause extensive alterations in the cell envelope and cell wall protein, especially the outer membrane of *P. gingivalis* [78]. Kuboniwa et al., 2006, showed that 84 proteins of cell envelope were detected during the co-infection, out of which 40 showed reduced abundance during the three species community formation [78,79]. Some of the common proteins affected during the co-infection include proteins regulating thiamine, cobalamin, and pyrimidine synthesis; OmpH proteins, two lipoproteins, MreB protein (a bacterial actin homolog) that play an important role in determining cell shape; and DNA repair proteins. The HmuR, a TonB dependent outer membrane receptor, was up-regulated in the community with an increase in protein synthesis. Some of the other encoded proteins that affected by this interaction include proteins for maintenance of cell wall integrity (murE), intercellular signaling (cbe), regulation of redox state (spxB and msrA), ribosomal proteins and translation elongation and initiation proteins. A decrease in vitamin B1, B 12, Biotin, pyrimidine synthesis was also observed during this co-infection [78,79] A community derived *P. gingivalis* biofilm also showed a significant reduction in DNA repair proteins along with an up-gradation of DNA repair genes [80].

Apart from the up-regulation of these genes, *P. gingivalis* regulates the expression of Zinc finger E-box-binding homeobox (ZEB2), a transcription factor, which controls epithelial-mesenchymal transition and inflammatory responses within the periodontal tissues [80,81]. *P. gingivalis* mediated ZEB2 regulation occurs through pathways involving β-catenin and Forkhead box protein O1 (FOXO1). *S. gordonii* can antagonize ZEB2 expression, and thereby control *P. gingivalis* mediated inflammation response. Furthermore, *P. gingivalis* and *S. gordonii* coinfection is even found to enhance the expression of Protein Tyrosine Phosphatase (Ltp1) and AI-2 in the periodontal tissues [60–64,69,77,81–91]. The increased Ltp1 expression subsequently dephosphorylates *P. gingivalis* tyrosine kinase 1 (Ptk1) receptors and increases the transcription of Complementarity Determining Region (CDrR) to promote colony formation [68,82,83,91] (Figure 4). *S. gordonii* also inhibits the production of CdhR (Community development and hemin Regulator), a negative regulator of Mfa1, and increases the co-adhesion and co-aggregation of *P. gingivalis* with other microorganisms [60–62,84,85]. However, once the colony has been established, it dampens the micro-colony development by utilizing the Ltp1 receptors and reducing Mfa1 expression [90]. The Ltp1 phosphatase activity is also known to down-regulate the transcription of luxS and several other genes involved in exopolysaccharide synthesis and transport of nutrients [90] Ltp1 also promotes the transcription of the *hmu* operon involved in haem uptake and dephosphorylation of the gingipain proteases. The increased CDrR expression even help *P. gingivalis* to acquire nutrients such as peptides and heam from its environment at the times of crisis [92]. Since *P. gingivalis* is unable to synthesize its porphyrin, it relies on exogenous porphyrin and heam biosynthesis intermediates, from host sources and other bacteria. *S. gordonii* helps *P. gingivalis* to acquire heam by promoting met- Hemoglobin formation in the presence of Oxy-Hemoglobin. The heme-binding protein receptor (Hmu-Y) of *P. gingivalis’* captures and extracts the Iron III-Protoporphyrin IX using the Met-Hemoglobin obtained from *S. gordonii* [89–97]. Hmu-Y along with its cognate outer-membrane receptor, HmuR, aid in the transportation of the heam molecule through the outer membrane of *P. gingivalis*. This process of acquiring heam by *P. gingivalis*, also dependent upon the protein TonB, which helps to transduce energy for the passage of heam and other ligands into the periplasm. Furthermore, the Hmu-Y receptors help *P. gingivalis* to increase the extracellular polymeric substances (EPS) production that mediates cohesion among the microbial community and prevents injury from any external physical forces [90–97]. Under the iron-limited conditions, *P. gingivalis* expresses a haemophore-like protein, HusA, to mediate the uptake of essential porphyrin and support its survival within the host [92–94]. The Hmu-Y receptor of *P. gingivalis* can even interact with the Interpain-A (InpA) of *P. intermedia* and promote community development [90,98,99].

Additionally, since *P. gingivalis* is devoid of LuxI/LuxR-based signaling mechanisms, it utilizes the LuxI/LuxR receptors on *S. gordonii* to enhance the gene expression responsible for the acquisition of hemin, extracellular polysaccharide formation, and accretion into micro-colonies [91–94]. The LuxS in *S. gordonii* regulate the levels of the ‘Ssp’ adhesins and influence its ability to adhere to micro-colonies and autoaggregation in *P. gingivalis* [50].

Recently, two novel proteins named ‘internalin pro-Clptein (InlJ)’ along with the ‘expression of the short fimbriae and the universal stress protein UspA’ have been linked with monospecies *P. gingivalis* biofilms formation and initial attachment [100–106]. Furthermore, the loss of several genes has been observed within the *P. gingivalis* colonies to regulate the growth of the biofilm and promotes its chance of survival. Some of the inhibitors of homotypic biofilm accumulation include Caseinolytic Mitochondrial Matrix Peptidase Chaperone Subunit X (ClpXP) along with ClpC, and GalE (UDP‐galactose 4‐epimerase) have been found in P. gingivalis microcolonies [99,103–107]. *P. gingivalis* is also observed to cause lysine acetylation in colonies, and this is considered as novel mechanism of metabolic regulation, adaptation, and survival during infection [105]. The acetylation of RprY, a response regulator of *P gingivalis* during the oxidative stress, has been considered a key factor regulating gene expression. Lysine acetylation reduces the promoter DNA binding ability of RprY, which consequently alters the gene expression of the host [105–107].

Apart from regulating the genetic expression and release of important proteins, *P. gingivalis* can even facilitate the growth of other bacterial species by acting as a ‘bridging species’ [99,108,109]. *P. gingivalis* facilitates the entry of pathogens into deep periodontal pockets. Studies have proven that the outer membrane vesicle of *P. gingivalis* (Omp A) facilitate co-aggregation and piggybacking of *T. denticola* [109–112]. Furthermore, *P. gingivalis* promotes the attachment of *Lachnoanaerbaculum. saburreum* to *T. denticola* by acting as a ‘bridge’ and facilitating complex microbial community formation [109–111]. The piggybacking of *T. denticola* and *Lachnoanaerbaculum saburreum* favors the migration of bacteria to the anaerobic sub-gingival niches and changing the ecology of the entire sub-gingival sulcus. The cooperation and co-existence of *P. gingivalis* with *T. denticola* can also increase the production of gingipains (Kgp, RgpA, HagA) and dentilisism from *T. denticola* [99]. The increased ‘gingipain’ production inhibits blood coagulation, escalates nutrient accumulation, and in turn favors attachment of *P. gingivalis* to the other microorganisms, epithelial cells, and fibroblast [113]. *P. gingivalis* is also known to ‘cheat’ its community as it retains some of the energy-consuming mechanisms that are discarded by other membranes of the community. This mechanism of retaining certain discarded features helps *P. gingivalis* to survive in times of crisis and support the growth and functioning of the entire microbial community [15].

An important metabolite that contributes regulate the metabolic interactions of *P. gingivalis* with other accessory pathogens and prevents its attachment to the host is arginine. Studies have shown that arginine deaminase (ArcA) of *Streptococcus cristatus* and *S. intermedius* impairs biofilm formation by inhibiting the production of FimA surface proteins [113–116]. The down-regulation of FimA catalyzes the conversion of arginine to citrulline. Reduced levels of extracellular arginine and/or citrulline accumulation inhibits FimA expression and biofilm formation [24,117]. An in vitro study on a mouse model has even confirmed that colonization of ArcA-expressing *S. cristatus* followed by *P. gingivalis* infection decrease the colonization of *P. gingivalis* [115]. Although the presence of ArcA from *S. gordonii* is yet to be confirmed, it is well characterized that *S. gordonii* ArcA has a well-developed arginine deaminase system (ADS), which catalyzes the intracellular conversion of arginine to ammonia and CO2, along with concomitant production of ATP [117]. Therefore, the distinctive response of *P. gingivalis* to *S. gordonii* is due to its ability to transform arginine into citrulline in an extracellular manner. Therefore, targeting ArcA surface proteins could form a ‘potential anti-biofilm agent to fight *P. gingivalis* infections’ [114–118]. Arginine and its derivatives also affect the interaction of *F. nucleatum* to other microorganisms in the biofilm [119]. *F. nucleatum* harbors an adhesin that is inhibited by arginine (RadD) [117,119]. Therefore high concentrations of arginine can inhibit cell-to-cell contact (i.e., coaggregation) between *F. nucleatum* and other bacterial species [120]. Another, novel cross-feeding interaction between *S. gordonii* and *F. nucleatum* is that involving ornithine, which is exported by the arginine-ornithine antiporter ArcD in the ADS of *S. gordonii*, has been reported [119]. In-vitro studies confirmed that ‘deletion of ArcD attenuates the accumulation of *F. nucleatum* in *S. gordonii* biofilm, while ornithine supplementation restored the bio-volume of *F. nucleatum* in mono-species and dual-species biofilms with the *S. gordonii* DarcD mutant’. This proves that ArcD-exported ornithine supports the growth of *F. nucleatum* and sustains the development of its biofilm. *F. nucleatum* is also known to increase the levels of ornithine decarboxylase (ODC), an enzyme responsible for the conversion of ornithine/arginine to putrescine, in the community biofilms formed with *S. gordonii* [37]. *F. nucleatum* utilizes the ornithine released by *S. gordonii* ArcD as a substrate of ODC and enhance the overall community development by mediating cross-feeding of ornithine, and reinforcing coaggregation between *S. gordonii* and *F. nucleatum*. These findings suggest that sustained delivery of ornithine from accessory pathogens induce a state of dysbiosis, by sustaining the growth of the entire microbial community [116]. Furthermore, *S. gordonii* produces a distinct pattern of protein in communities with *F. nucleatum* or *P. gingivalis*, especially with the ADS component enzymes ArcA, ArcB (catabolic ornithine carbamoyltransferase), ArcC (carbamate kinase), and ArcD has been observed [61–68,115,116,118]. The levels of ArcA, ArcB, and ArcC of S. gordonii were reduced in community biofilms formed with *P. gingivalis* as compared to mono-species biofilms. On the other hand, the interaction of *S. gordonii* with *F. nucleatum* showed a marked increase in the levels of ArcA, ArcB, and ArcC despite a significant reduction in the level of ArcD in *S. gordonii* [62–65,121].

***2b. Persistence and survival in the host cells by facilitating tissue invasive and immune modulation strategies***

### Neutrophil dysfunction and increase cytokine production

**Figure 6.**
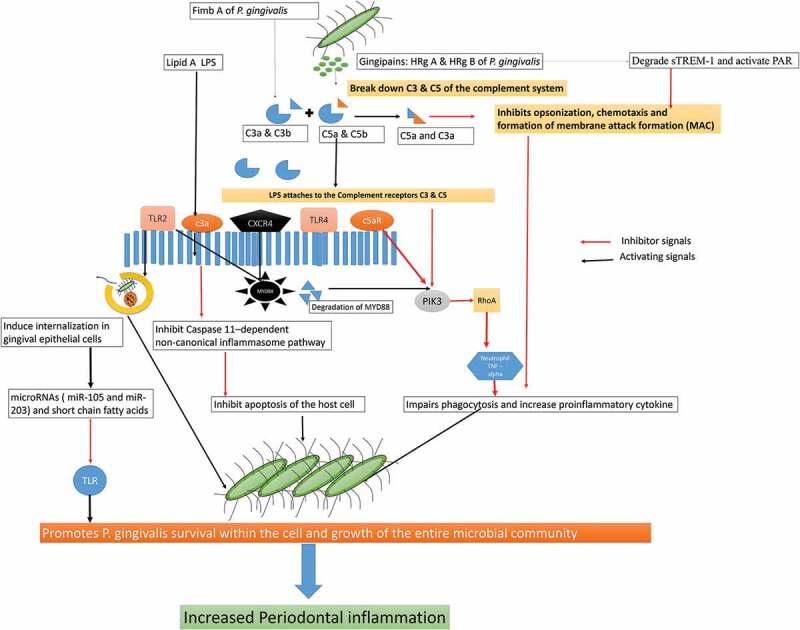
**Schematic representation Immune response pathways triggered by the activation of TLR2/TLR4/CXCR5/C5aR receptors by gingipains P. gingivalis**: The gingipains of P. gingivalis degrade the C5 and C3 from the complement system and degrade them C5a and C3a. The C5a interacts with the receptors on the neutrophils, epithelial cells, and endothelium in the host to Impairs phagocytosis and increase proinflammatory cytokine. C3a also inhibits the caspase 11–dependent non-canonical inflammasome pathway and prevents the apoptosis of the cell and allows P. gingivalis to use the host cell for its growth. The C5aR-TLR2 cross-talk activated by P. gingivalis pili induced degradation of MyD88 in neutrophils. In the absence of MyD88, the co-association of C5aR-TLR2 promotes P. gingivalis infection v activation of the TIRAP-dependent PI3 K signaling pathway. This, in turn, causes an inflammatory cytokine TNF-α along with inhibition of RhoA activation and actin polymerization that impairs the process of the maturation of phagosomes and P. gingivalis phagocytosis [Abbreviation: CR- complement receptors; TLR- Toll-like receptors; CXCR4: C-X-C chemokine receptor type 4; cAMP: cyclic adenosine monophosphate; iNOS: inducible nitric oxide synthase; Mal: MyD88 adapter-like; p38MAPK: mitogen-activated protein kinase p38; PKA: protein kinase A; PI3 K: phosphoinositide-3-kinase; RhoA: Ras homolog gene family, member A; sTREM-1 – sTREM-1 – salivary Triggering Receptor Expressed on Myeloid cells 1ʹ Protease-activated receptors [PAR]-2].

**Figure 7.**
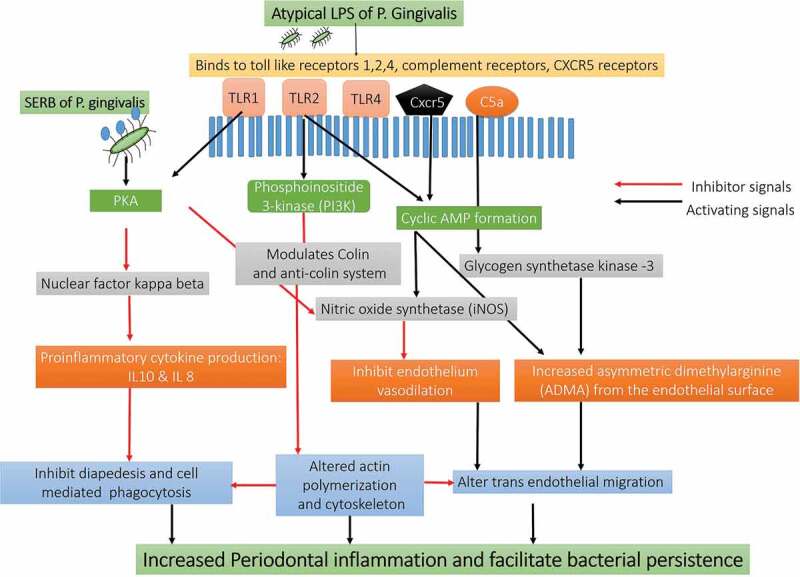
Schematic presentation of the immunological pathways triggered by LPS of P.*gingivalis.*

Apart from inter-bacterial interaction, *P. gingivalis* survives and persist within the host by evading the immune response and promoting cellular invasion [10–16,26,27,52,121]. The type II fimbriae of *P. gingivalis* adheres to the α5β1-integrin in the gingival epithelium [34,52,53,121–130] The fimbriae-integrin binding rearranges the actin cytoskeleton and exploits the cellular endocytosis to get internalized by encapsulation within an early endosome [25–27,46]. Upon entry into the gingival epithelium, *P. gingivalis* accumulates around the nucleus and remains viable before disseminating into the other parts of the cells [128–130]. It rapidly locates itself at the most anoxic part of the cytoplasm and utilizes the cellular machinery for its growth and survival. It even remodels the cytoskeletal microtubule dynamics of the cell by interacting with the integrin-dependent signaling pathway and actin molecules. The α5β1-integrin on epithelial cells is correlated with the S phase of the cell cycle, and *P. gingivalis* persistence may be associated with the ability to preferentially target dividing cells [47]. *P. gingivalis* is also associated with degradation of focal adhesion kinase and paxillinin the host cells, which could be linked with periodontal tissue degradation, poor wound healing, and regeneration in periodontitis [57,58]. The altered cytoskeleton disrupts the morphology and function of the actin molecule and allows further bacterial entry [128,131]. Once it exits from the invaded cells, it subsequently infects the ‘neighboring gingival cells’ that further invasion and persistent within the host [128]. *P. gingivalis* also release a functionally versatile compound, known as Serine phosphatase protein (SerB), which can modify the function of the actin molecule [131–137]. SerB can dephosphorylate the actin-depolymerizing molecule ‘cofilin’, a major actin-binding protein that regulates nuclear translocation, mitosis, chemotaxis, and actin-mediated endocytosis (Figure 6 & Figure7). SerB even alters the functioning of the actin microfilament and stability of the microtubules [96]. Therefore, SerB inactivated cofilin favors *P. gingivalis* invasion and promotes its survival within the cell [138–153].

The atypical LPS of P. gingivalis interacts with the TLR2/TLR4/CXCR5/C5aR receptors on the host cells to activate Phosphoinositide 3-kinase (PI3 K), cyclic AMP and PKA. The C5a-C5aR activation mediated by P. gingivalis gingipain-degradation of C5 synergistically enhances the production of cAMP. The combination of pili and CXCR4 helped maximize cAMP production via C5a-TLR2 cross-talk. The continuous increase in cAMP activated PKA to reduce macrophage-forming NO and destroying the bactericidal function and possible therapeutic targets. The increased cyclic AMP inhibits the release of nitric oxide that further impairs the process of transendothelial migration, chemotaxis, and phagocytosis. Arg-specific gingipains and SerB protein of *P. gingivalis* alters the process of actin polymerization. The altered actin affects the process of phagocytosis along with promoting the development of the pathogenic species that can indirectly increase the inflammatory process in the periodontal tissues [Abbreviation: CR- complement receptors; TLR- Toll-like receptors; CXCR4: C-X-C chemokine receptor type 4; cAMP: cyclic adenosine monophosphate; iNOS: inducible nitric oxide synthase; Mal: MyD88 adapter-like; p38MAPK: mitogen-activated protein kinase p38; PKA: protein kinase A; PI3 K: phosphoinositide-3-kinase; RhoA: Ras homolog gene family, member A]

Apart from using the host cell to gain entry, inactivation of cofilin by SerB impairs the process of phagocytosis, chemotaxis, and transendothelial migration of neutrophils [134–144]. It is also known to upregulate P-21 Activated Kinase (PAK) and Rho-Associated-Kinase (ROCK) receptors that indirectly increase pro-inflammatory cytokines production in the host [90–92]. SerB dephosphorylates the serine residues of the Nuclear factor-kappa Beta (NF-κB) and inhibits CXCL8 induced IL8 production [15,93–98,154-158]. Reduced IL8 levels impair the process of phagocytosis and allow *P. gingivalis* to use the epithelial cells as an autophagosome for its growth and survival [96,149–157]. The SerB even remains functionally viable within the cells by interacting with other bacteria proteins. For example, SerB of *P. gingivalis* binds to the Heat Shock Protein (HSP) and GAPDH (glyceraldehyde 3 phosphate dehydrogenase) of *Streptococcus oralis* within the periodontal tissues to increase the inflammatory response [80,83,100] *P. gingivalis* also inhibit, TLRs, E-selectin and intercellular adhesion molecular (ICAM) expression on the endothelial cell surface and binds to the non-chemotactic methionyl peptides receptor to inhibits the neutrophil chemotactic gradient, leukocyte adhesion, and recruitment of neutrophil to the site of inflammation [150–160]. The reduced chemotaxis decreases the rate of phagocytosis and facilitates the growth of the entire microbial community. The activation of TLR2 is required for bacterial persistence and its deficiency has been linked with inhibition of alveolar bone resorption [161]. Apart from SerB, lipopolysaccharide (LPS), FimA fimbriae, Lipoteichoic acid of *P. gingivalis* modulate the immunoinflammatory response by inhibiting the production of antimicrobial molecules, complement system, and recruitment of leukocytes and favoring colonization of microorganisms in the periodontal tissues [95–98,100,151,155,160–162] *P. gingivalis* release of various antimicrobial peptides (βeta defensins 2), activate the coagulation cascade and the kallikrein/kinin cascade to increase the inflammation in the host [150,160].

The LPS of *P. gingivalis* (4 acyl mono-phosphorylated lipid A moiety) with its two isoforms (Penta-acylated LPS 1690 and Tetra-acylated LPS 1435/1449 is even responsible for the dual inflammatory response by modulating the TLR4 in the periodontal tissues [23,50,150,155–163]. *P. gingivalis* LPS shifts its Penta-acylated 1690 isoform to tetraacylated 1435/1449 according to the changes in the environmental condition to survive and maintain its growth [158,159]. Moreover, the shift of LPS moiety disrupts the activity of the LPS binding protein (LBP) and downregulates LBP induced IL8 and IL6 production [157,164]. The shift of LPS moiety even modulates the complement system, subvert leukocytes phagocytosis, activate protease production, enhance virulence factors expression, and induce cytotoxicity [158–169]. Furthermore, the binding of LBP to the TLR4 receptor by using a membrane-bound or soluble cluster of differentiation molecule (CD14), triggers the release of various pro-inflammatory and anti-inflammatory mediators in the periodontal tissues [150]. The LPS of *P. gingivalis* even alters the functioning of cementoblast and osteoclast cells by modulating the RANKL/Osteoprotegerin (OPG) receptors. The LPS induced activation of RANKL receptors is associated with resorption and destruction of alveolar bone destruction [165,169,170].

*P. gingivalis* LPS also activates the TLR 2-CXC chemokine receptor 4 (CXCR4) and Complement receptors (C5aR) on the neutrophil surface [150–153,170]. The Arg-specific gingipains (RgpB and HRgpA) co-activate and mediate the cross-talk between complement receptor (C5aR), TLR2, and CXCR4 [157]. TLR2-C5aR activations increase the ubiquitin via E3 ubiquitin ligase Smurf1 dependent proteasomal degradation and ubiquitination of Myeloid differentiation primary response gene 88 (MyD 88). The inhibition of MyD 88 receptor blocks the signals from the TLR2-PI3 K pathway and activate IL1 receptors with increased production of IL 1 [95,100,110,156–159]. The crosstalk between C5aR with TLR2 activates the PI3 K signaling pathway and indirectly increases the inflammatory response [36,126,153]. The PI3 K activation prevents phagocytosis, inhibits RhoA activation, and actin polymerization. The inhibition of TLR2-PI3 K pathway even impairs the process of phagocytosis by inhibiting the RhoA GTPase dependent actin polymerization [95–98,100-102,161–163]. The inhibition of LR2/MyD88 signaling causes the death of infected neutrophils and blocks the process of phagocytosis in the host [29,96]. *P. gingivalis* can also inhibit all the components of the complement system (classical, alternative and lectin pathways) and degrades the byproducts of the complement system [139,163] *P. gingivalis* inhibits the Mannose-Binding Lectin (MBL), Ficolins (FCN), C3, C3b, and C4 components of the complement system and prevent the formation of the ‘Membrane Attack Complex’ (MAC) [161,163–168] The inhibition of MAC and other components of the complement system favors *P. gingivalis* to evade the complement-mediated phagocytosis and survive within the host [139–143]. Moreover, *P. gingivalis* utilizes its gingipain to entrap the circulating C4b-binding protein and defend itself from being phagocytized by the molecules of the complement system [109,141]. The Arg specific gingipains even possess C5 convertase like properties that increase the concentration of C5a in the gingival crevicular fluid [108]. The C5a binds to the C5a receptor (C5aR) on the leukocytes and impair leukocyte killing capacity [151,170]. The cross-talk between upon *P. gingivalis* with TLR2-CXCR4 receptors causes a sustained release of cAMP [171]. The increase in cAMP production weakens the macrophages induced nitric oxide synthase [iNOS] dependent phagocytosis and facilitate the growth of the entire microbial community (Figure 7) [20,114,126]. The increased c-AMP production along with a decrease in the IL10 levels reduces the production of nitric oxide (NO) from the endothelial cells. *P. gingivalis* also evade the nitric oxide synthetase expression by modifying the structure of the Lipid A moiety of its LPS that indirectly inhibit TLR4 activation [15,21]. Additionally, RgpB and HRgpA gingipains of *P. gingivalis* along with karilysin enzyme secreted from *Tannerella forsythia* and InpA molecules from *P. intermedia* activate the release of C5a from C5 and degrade the central component C3 and Immunoglobulin G [172]. This synergistic interaction of gingipains with Interpain A and karilysin weakens the host immune response and allows persistence of the microbe within the host [90].

The LPS of *P. gingivalis* also increases the production of thrombospondin-1 (TSP), an extracellular matrix protein secreted by monocytic cells in the periodontal tissues. TSP stimulates the movement of the macrophages and increases the macrophage-mediated inflammation in the periodontal tissues [173]. Additionally, *P. gingivalis* increases the production of IL17 to synergistically enhance the production of TSP1 and plasminogen activator inhibitor type I in the gingival and periodontal tissues. The increased production of TSP1 and plasminogen activator inhibitor type I contribute to the persistence of the microorganisms within the host that leads to the progression and chronicity of periodontal disease [174,175]. *P. gingivalis* persistence within the gingival epithelial cells is known to induce the production of several microRNAs, such as miR-105 and miR-203 and short-chain fatty acids. The miR-105 and miR-203 can suppress TLR2 function and inhibit the release of Suppressor of Cytokine Signaling 3 (SOCS3) and SOCS6 respectively [148,149]. The long-term cohabitation of *P. gingivalis* within the host cell favors the establishment of an ‘inter-kingdom’ whereby it impairs the important defense mechanisms of the host (Figure 6) [171,176,177].

Recently, *P. gingivalis* is found to increase the production of butyrate and propionate and decrease the release of cytokine-induced neutrophil chemoattractant (CINC) 2αβ, another powerful chemoattractant and inhibitor of chemokines production [132,175]. The outer membrane components of *P. gingivalis* is proven to possess ‘porin-like activity’ that can depolarize the electrochemical potential on the neutrophil membrane and prevents its migration in response to the chemotactic stimuli [132,171]. A study done by Chen et al. (2011) showed that the outer membrane protein (PG0027) is ‘essential for the O-deacylation of LPS, secretion of gingipain to the cell surface, and attachment of *P. gingivalis’* to host cells [171]. The hemaagglutinins and gingipains released from *P. gingivalis* help to create a nutrient-rich but oxygen-deficient environment that protects *P. gingivalis* from the immune response of the host and promotes its survival [22,113]. *P. gingivalis* along with *T. denticola and T. forsythia* degrade and inactivate various antimicrobial molecules and enzymes secreted by the neutrophils to survive in the inflammatory milieu [177–180]. Recently, the gingipains from *P. gingivalis* are found to degrade the salivary Triggering Receptor Expressed on Myeloid cells 1 (sTREM-1) on the neutrophils and impede the process of phagocytosis and chemotaxis (Figure 6). *P. gingivalis* utilize its Arg-gingipain and sTREM1 receptors to create an inflammation rich environment for its acquiring nutrients. However, when the inflammation starts to compromise its existence, *P. gingivalis* changes from its Arg-gingipain to Lys-gingipain and attenuates the periodontal inflammation. This ‘twin regulation’ of sTREM 1 by Lys and Arg gingipains is a unique mechanism adopted by *P. gingivalis* for its survival and persistence [179–183]. Nylund et al., (2017) evaluated the association of the Salivary TREM-1 (sTREM- 1)/its Ligand Peptidoglycan Recognition Protein **1** (PGLYRP1) in periodontitis patients with renal diseases and concluded that PGLYRP1 and sTREM-1 are strongly associated with increased proinflammatory cytokine production in patients with periodontitis [183]. Patients with deep and active periodontal pockets have more salivary sTREM-1 and PGLYRP1 concentration as compared to individuals with shallow probing depth. Further research has shown that *P. gingivalis* modulates sTREM-1 receptor by acting as a decoy receptor for TLR activation and inhibiting neutrophil migration. The simultaneous activation of TLR4 and sTREM1 synergistically increase the production of TNF αlpha, IL1β, and decreased the production of anti-inflammatory cytokines like IL10. The TLR-sTREM1 activation is also known to activate various receptors associated with increased production of proinflammatory cytokines such as IRAK-1 (IL1 R-associated kinases), MAPK, p38 MAPK, Jun N-terminal Kinase (JNK), PI3 K, ERK1/2, NF-kB (Figure 6 & Figure 7) [183]. TREM-1 molecule is known to activate nucleotide-binding oligomerization receptors (NOD1 and NOD2) and indirectly increase the activation of caspase and NF-kB receptors that can exaggerate the production of proinflammatory cytokine in an autocrine manner [184].

### Inhibition of apoptosis increased oxidative stress and activation of the inflammasome

*P. gingivalis* is also known to inhibit the apoptotic pathways of the infected host cell and utilizes the cellular machinery for its survival. The inhibition of apoptosis contributes to the chronicity of the periodontal disease by promoting the growth of other bacterial species (Figure 6) [17,91]. The LPS of *P. gingivalis* activates caspase 11 dependent non-canonical inflammasome and initiates the process of pyroptosis and lytic cell death within the macrophages [135,184–188]. *P. gingivalis* LPS, specifically the O-antigen region and HmuY is known to affect the ‘viability and apoptosis of gingival epithelial cells’ [54,62,155,187–190]. The HmuY protein of *P. gingivalis* can induce apoptosis in the gingival epithelial cells by increasing FAS Ligand expression and NFκB receptor activation. The Fas ligand, also referred to as CD95 L or CD178, is a type-II transmembrane protein belonging to the TNF family, which initiates the process of apoptosis. A study by Meghill et al., 2019 elucidates the underlying mechanisms by which *P. gingivalis* manipulates dendritic cell signaling to perturb both autophagy and apoptosis. The results of their study showed that the minor (Mfa1) fimbriae of *P. gingivalis* induce Akt nuclear localization and activate the Akt/mTOR axis required for autophagosome formation and maturation [190]. *P. gingivalis* also increase the mitotic cell cycle and suppress apoptosis by inhibiting Janus A Kinase (JAK), Phosphoinositide 3 Kinase (PI3 K), Signal Transducer of Activation (STAT), alpha-serine/threonine-protein kinase (Akt) and purinoceptor (P2X7) pathways (Figure 7) [91] Furthermore, *P. gingivalis* reduce the renewal capacity of cells and inhibit apoptosis by activating p38, mitogen-activated protein kinase (MAPK), and extracellular-signal-regulated kinase (Erk1/2) pathways [184–186] The activation of p38, MAPK, and Erk1/2 pathways decrease the expression of cyclin D and inhibit cellular proliferation by arresting the cell cycle at the G1 phase [72,73]. Studies have confirmed the deregulation of apoptosis-related genes, such as Bax, Bcl2, Nlrp3, or Smad2, in the gingival tissues of patients with periodontitis [181,189]

*P. gingivalis* has also confirmed to possess cell-specific modulation of apoptosis receptors and signaling pathways such as Apoptotic protease activating factor (APAF 1), B-cell lymphoma Associated X (Bax1) and Caspase and reduce B-cell lymphoma (BCL 2) in the epithelial cells and fibroblasts [184]. In the epithelial cells, *P. gingivalis* blocks the epithelial cell death by decreasing the APAF-1 expression, reducing the enzymatic activity of caspase enzyme, and increasing the expression of X-linked inhibitor of apoptosis protein (XIAP). However, in the fibroblast, *P. gingivalis* stimulates the APAF-1 pathway and reduces XIAP expression, increases caspase expression and apoptosis. The oligomerization of APAF1, induced by its binding to cytochrome C, helps apoptosome formation, a structure that recruits and activates a caspase initiator, caspase 9. The activation of caspase 9 cleaves and activates caspase effector caspase 3 and caspase 7, leading to apoptosis [184]. The increased caspase expression following *P. gingivalis* infection increases the process of pyroptosis, hypoxia, and exaggerate the inflammatory burden in the periodontal tissues and systemic circulation [190–195].

*P. gingivalis* can even inhibit *F. nucleatum* induced activation of the NLR family pyrin domain containing 3 (NLRP3) inflammasome and modulates the NLRP3 inflammasome cytokine secretion from the macrophages [189]. NLRP3 inflammasome is associated with the release of cytokines (IL1b and IL18), apoptosis, phagocytosis, and pyroptosis. NLRP3 regulates the process of apoptosis in the osteoblasts cells infected with *P. gingivalis* and *A. actinomycetemcomitans* [188]. Additionally, *P. gingivalis* interacts with the CR3 receptors on the macrophages and decreases the IL12p70 induced bacterial clearance by the macrophages [190–193]. The enzyme sialidase in *P. gingivalis* has been lately discovered as novel mechanisms to inhibit macrophage activity. Studies have shown that sialidase-deficiency in *P. gingivalis* can attenuate CR3 activation in macrophages, reduce inhibition of lncRNA GAS5, with less miR-21 and more IL12 production. Inhibition of sialidase in *P. gingivalis* would render *P. gingivalis* more easily cleared by macrophages [193]. The compromised macrophage function impairs the process of antigen presentation, lymph node activation, and delays the onset of cellular immunity in the host [189].

Additionally, *P. gingivalis* increases oxidative stress and extracellular ATP production by activating the pannexin-1 receptors (P2X4, P2X7) and NADPH-oxidase family members (NOX 4) [194–199]. NOX4 is a transmembranous located enzyme in the endothelial cells, fibroblasts, keratinocytes, and osteoclasts of the periodontium [197–199]. The ATP stimulation through ligation of P2X7 receptors synergistically increases the production of NADPH oxidase and exaggerate the mitochondrial ROS production in the periodontal tissues [147].

Studies have even confirmed that *P. gingivalis* infection increases the production of 8 hydroxy 2ʹ deoxyguanosine, superoxide anions, matrix metalloproteinases, caspase-3, catalase enzyme in the host cells [192,197]. *P. gingivalis* protect itself against the extracellular ATP and oxidative stress by releasing a unique enzyme known as nucleotide-diphosphate-kinase (NDK) (Figure 1) [147]. NDK cleaves the extracellular ATP molecules and down-regulates the P2X7/pannexin 1 receptor that is activated by autocrine action of ATP, within the host [200]. The NDK from *P. gingivalis* accumulates in the cytoplasm and is subsequently carried along myosin-9 filaments and actin filaments to the host cell periphery [150]. Upon translocation to the extracellular environment through the formation of the P2X7/pannexin 1 channel, NDK hydrolyzes ATP, and decrease the ATP-induced IL-1β release [92,150,152]. Additionally, the NDK inhibits ATP and increase the reactive oxygen species (ROS) production that helps in bacterial persistence [150–152,201-203]

Furthermore, it has been observed that even though *P. gingivalis* increase the oxidative stress in the host, it remains unaffected by the ROS due to the release of various endogenous antioxidants, like thiol peroxidase, glutathione, superoxide dismutase, rubrerythrin, in and around its cell surface [18,147,180–183,200,204,205]. The endogenously produced antioxidants protect *P. gingivalis* from the ROS mediated injury and allow it to survive within the host. The increase in the levels of glutathione suppresses the mitochondrial-mediated intrinsic cell death and protects the bacteria against the reactive oxygen species (ROS) that help *P. gingivalis* to survive within the host [18,147]. *P. gingivalis* selectively inhibits the macrophage-mediated cytokine production without affecting the production of T cell-mediated chemokines [206] The selective inhibition of the inflammatory process allows it to acquire the essential nutrient derived from the dead bacteria and tissue breakdown products for its growth and provide a competitive advantage to *P. gingivalis* during the inflammatory process [18,20,119,126,188].

### Alteration of T cell function with activation of Th17 cell

Another important mechanism by which *P. gingivalis* impairs the host immune response is by altering the T cell function and activation of Th17 mediated cytokine production [201–203,205-208]. It has been observed that *P. gingivalis* can induce TLR2-dependent IL-10 production that ‘leads to inhibition of IFN-γ production by CD4+ and CD8 + T cells [209]. *P. gingivalis* down-regulates IL12 secretion and increases the release of Interferon-alpha (IFN-alpha) from the CD4 Th1 cells. The decreased levels of IL12 and increase the production of IFN-alpha shifts the immune response from Th1 to Th2 [14]. The outer membrane proteins of *P. gingivalis* is found to activate the Th1 cells that in turn increase the production of proinflammatory cytokines [210]

Furthermore, *P. gingivalis* interacts with the dendritic cells of the host and favors the production of Th17 related cytokines such as TNF*α*, IL8, CXCL8, IL17, IL1β, IL6, IL23, IL12p40, IL21, IL3, granulocyte colony-stimulating factor (GCSF), etc. from both immune and non-immune cells of the host [147–149,202,203,206–210]. The increased production of IL1, IL 6, IL 21, IL23, TGF βeta amplifies and stabilizes the Th17 cell differentiation and favors the onset of dysbiosis and periodontal inflammation. The activation of Th17 cells increases the levels of IL1β, IL6, and TNFα in the periodontal tissues, which is associated with increase bone and soft tissue destruction in the periodontal tissues [210]. IL17 activates the RANKL receptor on the osteoblasts and fibroblasts and induce bone resorption by expressing the ‘osteoclast activating factor’ on the alveolar bone [210]. Furthermore, the arg-gingipain from *P. gingivalis* is known to modulate and increase the production of proinflammatory cytokines by acting on novel receptors sites like protease-activated receptors [PAR]-2 and soluble triggering receptor expressed on myeloid cells [sTREM-1] [211]. The activation of PAR-1, PAR-2, and PAR-4 induce CD69 and CD25 expression in CD4^+^ T cells that in turn increase the production of IL17 [211–215]. *P. gingivalis* also interacts with the NF-κB and RAR-related orphan receptor (ROR) transcription factor on the alveolar bone and increases the production of IL17 mediated bone resorption [213].

### 2 c. ‘Fratricide and Programmed Cell Death’ of other bacterial species

*P. gingivalis* facilitates ‘Fratricide and Programmed Cell Death’ of other bacterial species within the biofilm to increase the inflammatory response and promotes its survival [60,216] Fratricide is the pathogenic process by which *P. gingivalis* kills and obtain DNAs fragments from noncompeting host cells and microbes by inducing their death [216,218–221] During the process of programmed cell death, *P. gingivalis* promotes a fraction of the microbial population to perform a self-sacrificing ‘suicide’ and discharge its nutrients and extracellular DNA fragments into the environment. The discharged nutrients and extracellular DNA fragments are utilized by *P. gingivalis* and other members of the microbial community for their growth. The process of ‘Fratricide and Programmed Cell Death’ is tightly regulated by a pattern of genes along with the presence of an intricate toxin and antitoxin system [216]. For example, when *P. gingivalis* strains are added to the biofilm containing *Streptococcus. mitis* and *Staphylococcus aureus*, cell death and altruistic suicide of microbial colonies take place [60] Some of the essential molecules that are linked with Fratricide and Programmed Cell Death include Choline Binding protein D (CbpD), Competence-induced bacteriocins (CibAB), Autolysin-Encoding Gene (LytA, LytC, LrgAB, LytR, and LytSR), CidABC operon, etc [220]. When *P. gingivalis* is added to biofilms containing *S. mitis*, apoptosis-like death with release of DNA fragments and production of transposase that marks the onset of programmed cell death have been observed [60]. *P. gingivalis* is even known to activate the bacterial apoptosis endonuclease (BapE) enzyme that can fragment the chromosomes by sequentially cleaving the supercoiled DNA. The DNA damage and induced chromosome fragmentation activate the process of apoptosis in the entire microbial community and intensify the process of dysbiosis and inflammation in the host [166–168].

## Conclusion

*P. gingivalis* is a potential and highly virulent periodontal pathogen that adopts intricate molecular mechanisms to interact with other members of the microbial community and host to pave its path of invasion, survival, and persistence. It adopts unique molecular and intricate mechanisms to survive and persist within the tissues and exaggerate inflammation and dysbiosis. *P. gingivalis* modulated the entire oral microbiome and creates an environment that not only favors its growth and survival but also facilitates the growth of many other commensal and pathobionts species. The interbacterial interactions, immune evasion, and tissue invasive properties help *P. gingivalis* to survive even in adverse environmental conditions and reach a distant organ system. However further research on how *P. gingivalis* invade the epithelial barrier of different organs and induce organ dysfunction is intriguing and needs more research. It is also necessary to explore if the altered immune response by *P. gingivalis* considerable enough to acquire other opportunistic infections and systemic diseases. More research is necessary at the genomic level to understand and discover the transcriptomes, nucleotide, and metabolites affected in the host by *P. gingivalis* with the host and other members of the microbial community upon invasion. It is also necessary to explore if these immunologic mechanisms form a link between *P. gingivalis* and the development of various cancers. Furthermore, it is also critical to explore if any of these cellular and molecular mechanisms are associated with the development of emerging antimicrobial resistance of periodontal pathogens to conventional antibiotics. There is a need to study the strain-specific variation of *P. gingivalis* on the modulation of host response and systemic inflammation. The paper is of paramount importance to researchers, oral biologists, microbiologists, immunologists, scientists, and pharmacologists across the globe to initiate further research and develop novel therapeutic modalities that can target the specific mechanism and prevent the onset of oral and systemic inflammation in the host
